# Supplementary material for: Somatic deficiency causes reproductive parasitism in a fungus
Source: Nat Commun. 2021 Feb 4;12:783. doi: 10.1038/s41467-021-21050-5 (PMC7862218; doi:10.1038/s41467-021-21050-5)
Supplement: Supplementary file 1 — Supplementary Information [file 41467_2021_21050_MOESM1_ESM.pdf]

Supplementary Information

"***Somatic deficiency causes reproductive parasitism in a fungus***"

Grum-Grzhimaylo et al.

1. **Supplementary Discussion**
2. **Supplementary Figures 1-9**
3. **Supplementary Tables 1-3**
4. **Supplementary References**

1. Supplementary Discussion

No temporal benefit of the Δ*so*-cheater: An alternative hypothesis that could explain a competitive benefit of Δ*so* is that Δ*so*-rich mycelia sporulate earlier than wild type. The rationale for this idea is that sporulation is induced when local resources are exhausted, which happens earlier in a more fragmented fungal colony. We tested and rejected this hypothesis (Supplementary Fig. 2). First, Δ*so* was not overrepresented during the earlier stages of sporulation relative to its frequency later on. Second, we did not find a relative benefit of Δ*so* nuclei in competition with an incompatible wild-type strain (Fig. 2b, Supplementary Fig. 8).

Frequency dependence of the benefit of the Δ*so*-cheater: Our finding that the Δ*so* cheater has a benefit in the heterokaryon, explains its initial selection, since a nucleus with a *de novo* fusion mutation will find itself in a heterokaryon. At this initial low frequency, mutant nuclei will have an increased likelihood to end in the spores of this heterokaryon. With increasing frequency, increasing numbers of spores will be homokaryotic for the fusion deficiency. Upon germination, a fraction of these will be fused with wild-type or heterokaryotic mycelia, thus forming new heterokaryons, and the rest will develop into homokaryotic mycelia that have low reproductive success. Figure 1a shows that Δ*so* has a benefit below ~30% when starting from homokaryotic Δ*so* and wild-type spores, and Figure 3b that it has a benefit up to a frequency of ~60% when starting from a heterokaryon. The difference implies that in addition to frequency dependence within the heterokaryon, there is also frequency dependence of the chance to fuse (Fig. 4b, c). This can be understood by considering the probability that a fusion mutant fuses with at least one wild-type individual. At low frequencies, the Δ*so* mutant is mostly surrounded by wild type, maximizing its chance one of these fuses with it. With increasing frequencies of the Δ*so* the proportion of wild-type contacts decreases thus reducing the chance that at least one of those fuses with a focal Δ*so*. Moreover, wild-type colonies increasingly become fragmented due to Δ*so* patches, and more Δ*so*-rich heterokaryons increasingly segregate into homokaryotic sectors, reducing connectivity between supportive somatic and reproductive structures. This provides a relative benefit to wild type, restricts the opportunities of Δ*so* to end in spores upon fusion with a wild-type colony and explains further reduction in overall spore yield.

Heterokaryon enforcement when measuring Δ*so*-cheater frequency in mycelium and spores: Supplementing or not supplementing the media affects heterokaryon maintenance. Supplementing the medium would not enforce it, while not supplementing the media would enforce it, because of deficiencies complementation between genotypes. The reason behind including these treatments was to test if non-enforced conditions would disrupt the heterokaryon by potential outgrowth of either of the two genotypes. We did not see that, as we could still detect both genotypes present in a chimera. Yet, at non-enforced conditions, the drop in frequency of Δ*so* in the mycelium was stronger, than at enforced conditions (Supplementary Fig. 9a). However, we saw almost identical Δ*so*-cheater dynamics between the two treatments upon sporulation (Supplementary Fig. 9b). Since non-enforced conditions resemble the conditions of the evolution experiment, we present the data coming from the supplemented conditions in the main text (Fig. 3).

2. Supplementary Figures

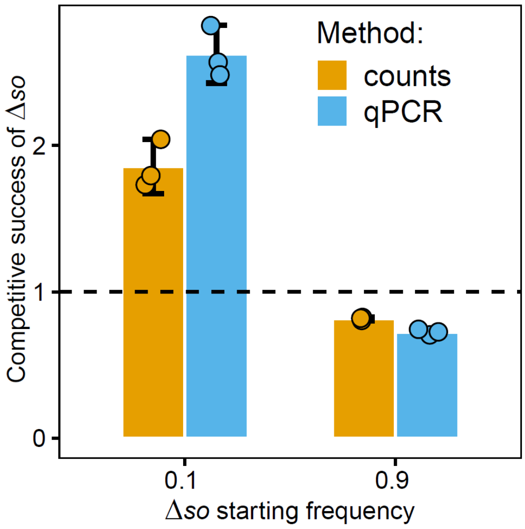

Supplementary Figure 1. Competitive success of Δ*so* against the wild type at low (~10%) and high (~90%) frequency as measured by phenotype counts and by qPCR. The competitive success of Δ*so* was calculated after 4 days of co-culturing (Δ*so* + wild type mixtures) as the frequency of Δ*so* after the competition divided by the initial frequency of Δ*so*. Phenotype counts and qPCR give conceptually similar results. Error bars are 95% confidence intervals around means calculated from three biological replicates. The replicate data points are overlaid as dots. Source data are provided as a Source Data file.

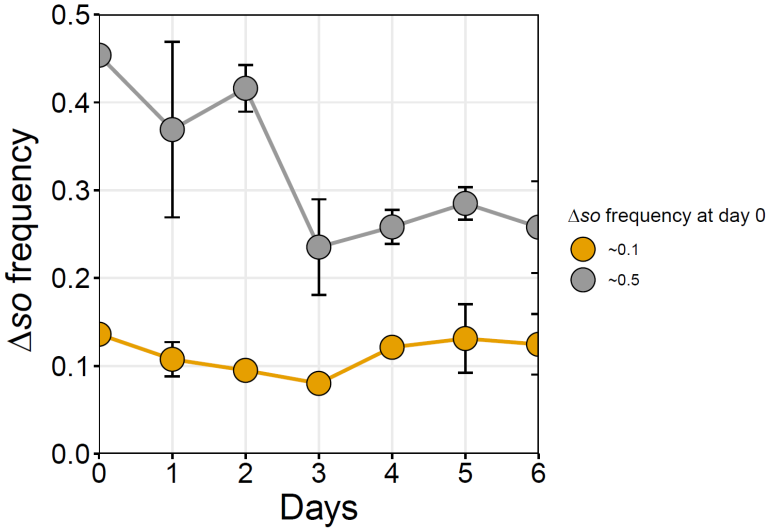

Supplementary Figure 2. No temporal benefit of Δ*so* against wild type. To follow the competitive benefit of Δ*so* in time, we sampled the competitions every day for six days. When started at ~50%, Δ*so* was losing to the wild type as competition progressed in time, in contrast to when starting at lower frequency (~10%). The frequency of Δ*so* was calculated using phenotype counts after plating the competition mixtures. Error bars are 95% confidence intervals around means calculated from three biological replicates. Source data are provided as a Source Data file.

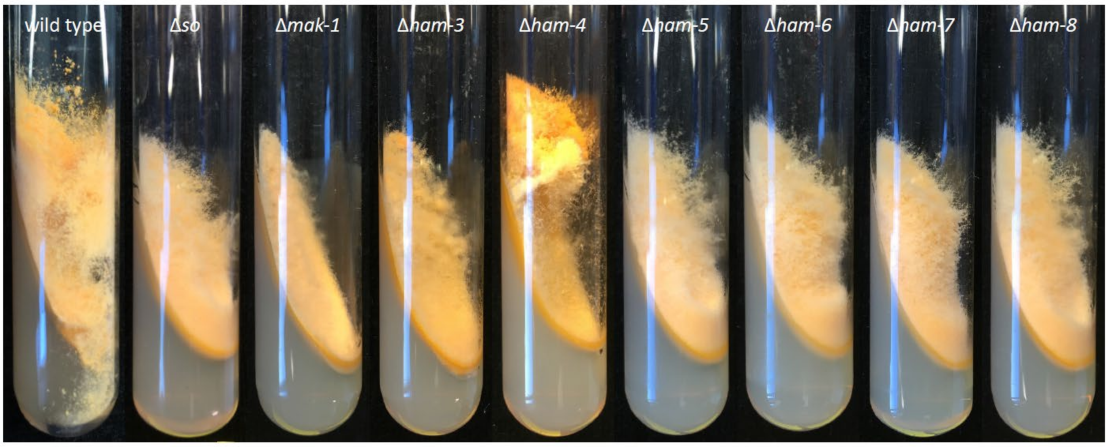

Supplementary Figure 3. Phenotypes of fusion mutants (and the wild type, for contrast) used in the study. 7-day old cultures on slanted VMM medium.

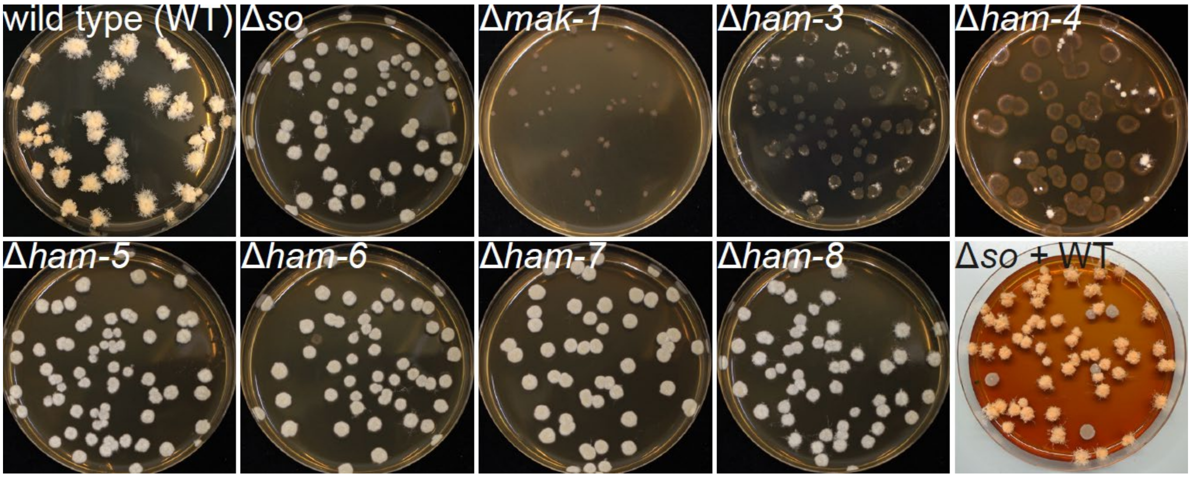

Supplementary Figure 4. Phenotypes of fusion mutants (and the wild type, for contrast) used in the study. 7-day old colonies on 9-cm Petri plates with sorbose-VMM (i.e. counting plates). The bottom-right plate shows a mixture of abundant wild type and low-frequency Δ*so*.

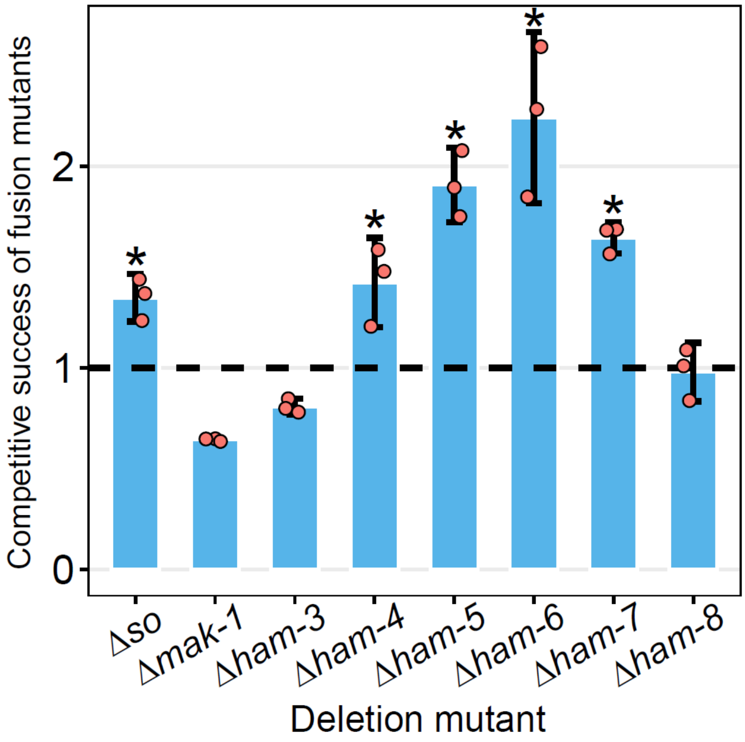

Supplementary Figure 5. Competitive success of fusion mutants at low starting frequency (~10%) against the wild type. The competitive success of fusion mutants was calculated using phenotype counts after 4 days of co-culturing (fusion mutant + wild type mixtures) as the frequency of fusion mutant after the competition divided by the initial frequency of fusion mutant. Error bars are 95% confidence intervals around means calculated from three biological replicates. Asterisks indicate statistically significant ($P < 0.05$) higher competitiveness than wild type. One-tailed Student's *t*-test: Δ*so*/wt: t-value = 5.7860, P = 0.0142975; Δ*mak-1*/wt: t-value = -85.1019, P = 0.999931; Δ*ham-3*/wt: t-value = -9.6499, P = 0.994715; Δ*ham-4*/wt: t-value = 3.7405, P = 0.0323107; Δ*ham-5*/wt: t-value = 9.5707, P = 0.00537052; Δ*ham-6*/wt: t-value = 5.7381, P = 0.0145270; Δ*ham-7*/wt: t-value = 16.1410, P = 0.00190842; Δ*ham-8*/wt: t-value = -0.2916, P = 0.600992). No adjustments were made for multiple comparisons. The replicate data points are overlaid as dots. Source data are provided as a Source Data file.

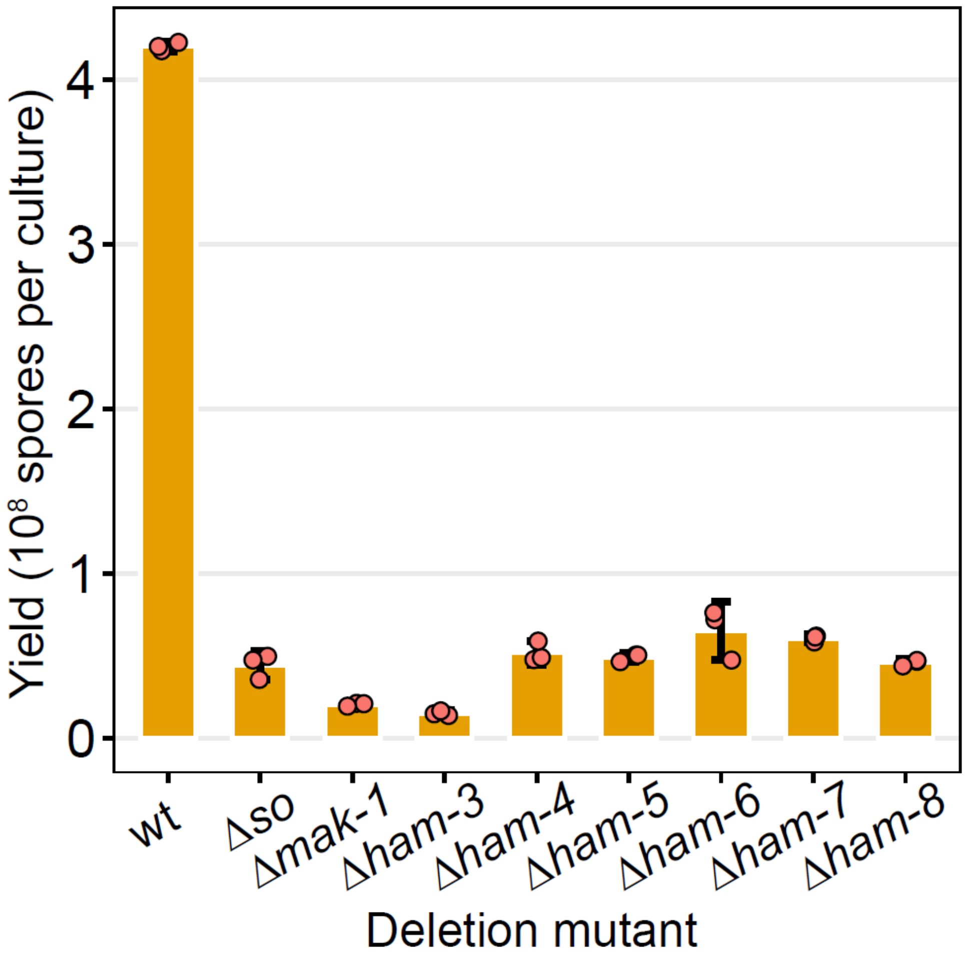

Supplementary Figure 6. Spore yield of fusion mutants in 4-d-old VMM slants (along with the wild type control for contrast). Error bars are 95% confidence intervals around means calculated from three biological replicates. The replicate data points are overlaid as dots. Source data are provided as a Source Data file.

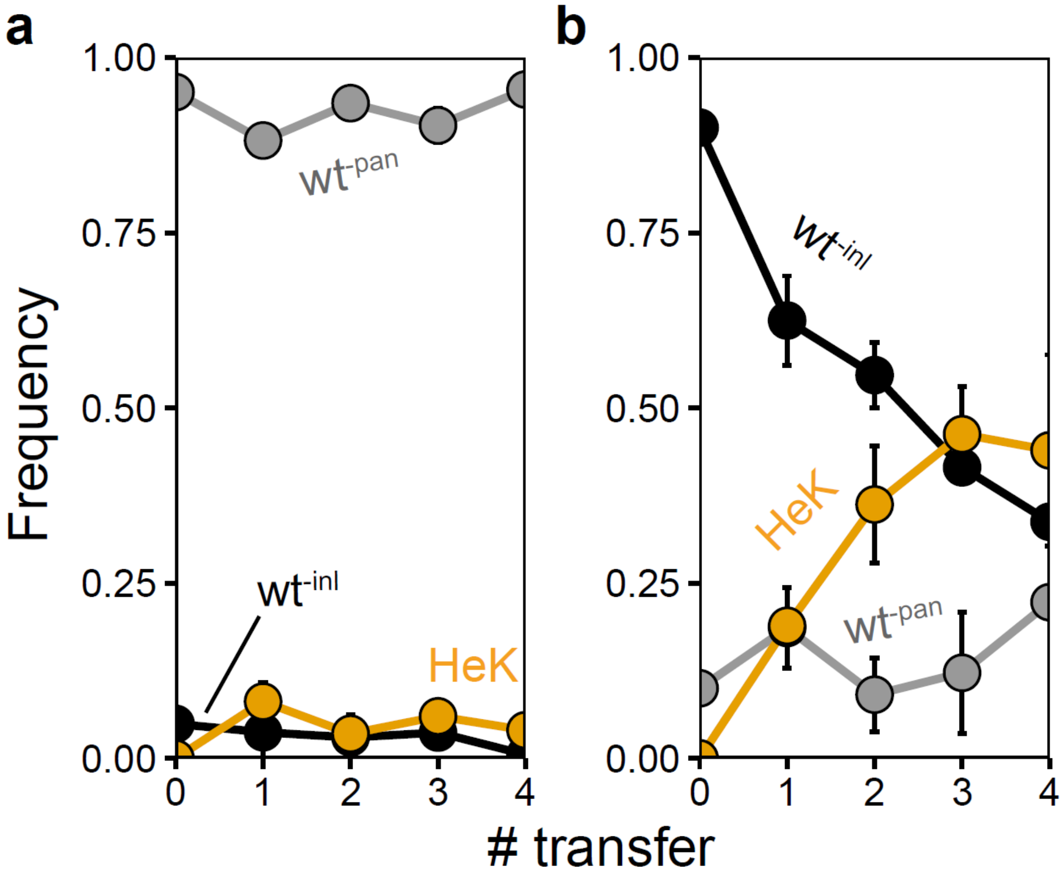

Supplementary Figure 7. The frequency of fusion between reciprocally labelled wild types. a, Transfer experiment with wild-type strains, at low starting frequency (~5 %) of inositol-deficient (*inl*-deficient) wild type. In this experiment we observed the strong effect of *inl* marker. **b,** Transfer experiment with wild-type strains, at low starting frequency (~15 %) of pantothenic acid-deficient (*pan*-deficient) wild type. Fusion readily occured between wild types. In both panels, the spore transfers were performed every 4 days with 1% of total spores. The frequency of genotypes was calculated using phenotype counts at every transfer on non-supplemented plates and supplemented with inositol or pantothenic acid. *inl*-deficient and *pan*-deficient strains are indicated in black and grey, respectively. The orange line shows the frequency of heterokaryons (HeK). In both panels, error bars are 95% confidence intervals around means calculated from three biological replicates. Source data are provided as a Source Data file.

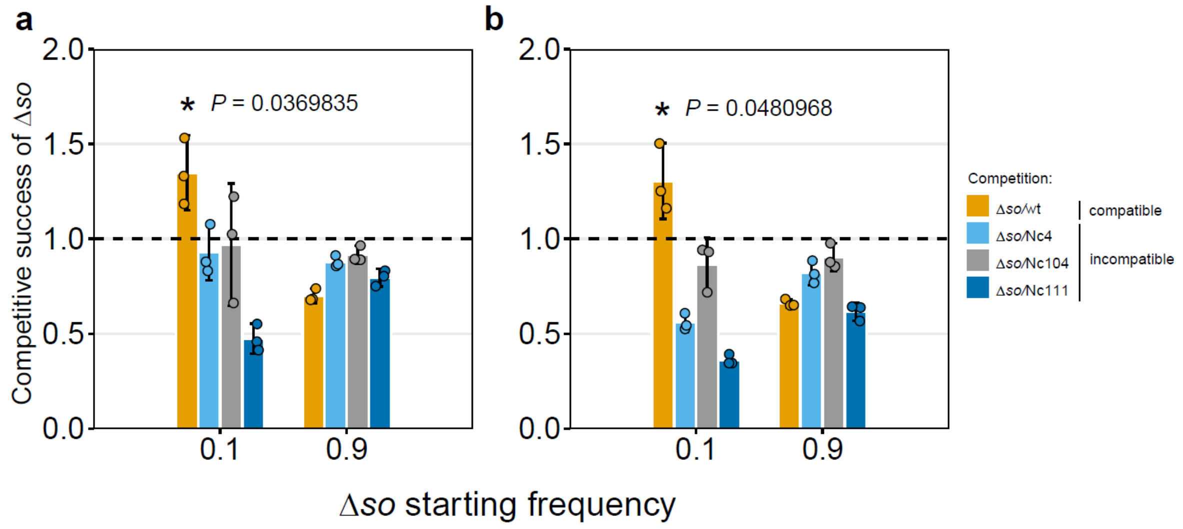

Supplementary Figure 8. Competitive success of Δ*so* against vegetatively compatible (wt) and incompatible (Nc4/Nc104/Nc111) wild types at low (~10%) and high (~90%) frequencies. a, The competitive success of Δ*so* was calculated using phenotype counts after 4 days of co-culturing (Δ*so* + wild type mixtures) as the frequency of Δ*so* after the competition divided by the initial frequency of Δ*so* (one-tailed Student's *t*-test, t-value = 3.469689, df = 2, *P* = 0.0369835). **b,** The competitive success of Δ*so* was calculated using qPCR (one-tailed Student's *t*-test, t-value = 2.986799, df = 2, *P* = 0.0480968). For details on qPCR method, see section "Frequency of Δ*so* cheater in heterokaryons" in Methods. Asterisks indicate significant difference of $P < 0.05$. In both panels, error bars are 95% confidence intervals around means calculated from three biological replicates. The replicate data points are overlaid as dots. Source data are provided as a Source Data file.

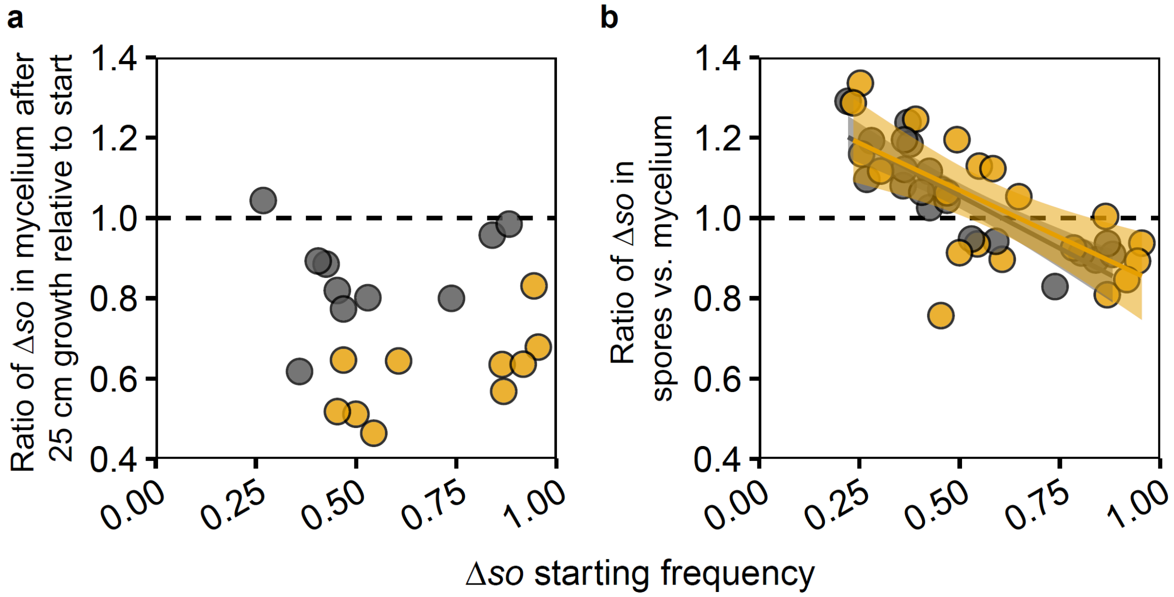

Supplementary Figure 9. How Δ*so* realizes a competitive benefit in chimeras. a, During linear mycelial growth of ten random heterokaryons, the frequency of Δ*so* decreased, irrespectively of the starting frequency. A condition not enforcing the heterokaryon formation (orange dots) showed a stronger reduction of Δ*so* during somatic growth relative to strains that enforce heterokaryon formation (grey dots). For enforced conditions (grey dots): one-sample one-tailed Student's *t*-test, t-value = -3.6702, df = 9, *P* = 0.002577; linear regression, F-statistic 1,8 = 0.5143, df=8, *P* = 0.4937. For not enforced conditions (orange dots): one-sample one-tailed Student's *t*-test, t-value = -11.692, df = 9, *P* = 4.803e-07; linear regression, F-statistic 1,8 = 5.309, df=8, *P* = 0.05014. **b,** In contrast, during sporulation of ten random heterokaryons, Δ*so* nuclei have a benefit over wild-type nuclei as long as starting frequencies remain below ~60%, irrespective of whether heterokaryon formation is enforced (grey dots) or not (orange dots). For enforced conditions (grey dots): linear regression, F-statistic 1,18 = 52.97, df=18, *P* = 9.176e-07. For not enforced conditions (orange dots): linear regression, F-statistic 1,18 = 15.71, df=18, *P* = 0.0009106. Twice as many data points on this panel (compared to **a**) is explained by using the end- and start-points of the same heterokaryon after its 25 cm linear growth for sporulation. Shaded areas around the trendlines are 95% confidence areas around the fitted lines. The frequencies Δ*so* in both panels were calculated using qPCR. Source data are provided as a Source Data file. For details on statistics, see section "Frequency of Δ*so* cheater in heterokaryons" in Methods.

3. Supplementary Tables

Supplementary Table 1. Raw reads and mapping statistics of the sequenced *Neurospora crassa* morphotypes.

Evolution line	Morphotype code	Raw reads (paired-end)	Trimmed reads (paired-end, length>=70,q>=30)	% trimmed	Mapped to reference genome (mapping q>=20, samtools flagstat: N = mapped - supplementary - secondary)	% mapped	Average coverage (only covered bases)
ancestor to lines 9-16	Nc152	16261518	12316616	24.26	12148931	98.64	41.6636
ancestor to lines 1-8	Nc159	16254600	12380134	23.84	12276868	99.17	41.3514
1	1t1	9685772	7413860	23.46	7356736	99.23	24.9313
	1t2	8114430	6986582	13.90	6791561	97.21	23.8172
2	2t1	9345284	7616010	18.50	7547200	99.10	25.6872
3	3t1	8134722	7148050	12.13	7059575	98.76	24.6833
	3t2	9729360	7819680	19.63	7742111	99.01	26.2718
4	4t1	8075234	7223682	10.55	7075664	97.95	25.0979
	4t2	9706892	7407036	23.69	7359402	99.36	25.1834
	4t3	8126006	7401912	8.91	7254109	98.00	25.7924
5	5t1	8131214	7324702	9.92	7227729	98.68	26.0144
	5t2	8132348	7256980	10.76	7169275	98.79	26.4282
6	6t1	9717026	7457396	23.25	7404300	99.29	25.5318
	6t2	8143338	7308602	10.25	7215427	98.73	25.7273
7	7t1	8142850	7318600	10.12	7216574	98.61	25.6734
	7t2	8142338	7207082	11.49	7117221	98.75	26.2664
8	8t1	8134808	7350744	9.64	7257171	98.73	25.8312
	8t2	8083878	7164486	11.37	7008072	97.82	24.7822
9	9t1	9762050	7679238	21.34	7614671	99.16	25.7705
	9t2	9762066	7666062	21.47	7601877	99.16	25.789
10	10t1	9745086	7723500	20.74	7648961	99.03	25.9672
	10t2	8138704	7358190	9.59	7264022	98.72	26.055

11	11t1	9604228	7439098	22.54	7380601	99.21	25.6529
	11t2	9748028	7820730	19.77	7746580	99.05	26.347
	11t3	9443604	7553618	20.01	7483980	99.08	25.3585
12	12t1	9769916	7449592	23.75	7395346	99.27	25.169
	12t2	9237560	7027008	23.93	6973514	99.24	23.8072
13	13t1	9588674	7455312	22.25	7385870	99.07	24.9664
	13t2	8136636	7340408	9.79	7254748	98.83	26.2485
	13t3	9572632	7541784	21.22	7466937	99.01	25.1583
	13t4	9741268	7545120	22.54	7471190	99.02	25.0438
14	14t1	9473952	7409758	21.79	7337594	99.03	24.7141
	14t2	8831642	6933110	21.50	6863054	98.99	23.2501
15	15t1	9757916	7599888	22.12	7532955	99.12	25.5648
	15t2	9721700	7291480	25.00	7240678	99.30	24.82
16	16t1	9520354	7047796	25.97	6994709	99.25	23.6181
	16t2	9358704	7244788	22.59	7170883	98.98	23.909
	16t3	9217438	6975760	24.32	6901601	98.94	21.9068

Supplementary Table 2. Strains of *Neurospora crassa* used in the study.

**Genotype**	**Gene(s)# affected**	**Obtained from FGSC (#), or from a cross in this study, or a reference**	**Internal code**	***het***
mat A		2489	147	Cde
mat a		4200	148	Cde
inl; *mat* A	NCU06348 (*inl*)	[1]	152	Cde
pan-2; *mat* A	NCU10048 (*pan-2*)	[1]	156	Cde
al-2; *pan-1*; *mat* A	NCU00585 (*al-2*); NCU08661? (*pan-1/pan-4*)	1425	004	cDE
inl; *mat* A	NCU06348 (*inl*)	538	104	CdE
al-2; *pan-1*; *mat* A	NCU00585 (*al-2*); NCU08661? (*pan-1/pan-4*)	2662	111	cde
Δ*fl*::*Hyg*^{r}; *mat* A	NCU08726 (*fl*)	Δ*fl*::*Hyg*^{r}; *mat* a x mat A	192	Cde
Δ*fl*::*Hyg*^{r}; *mat* a	NCU08726 (*fl*)	11044	166	Cde
Δ*so*::*Hyg*^{r}; *mat* A	NCU02794 (*so*)	11293	159=167	Cde
Δ*so*::*Hyg*^{r}; *mat* a	NCU02794 (*so*)	Δ*so*::*Hyg*^{r}; *mat* A x mat a	193	Cde
Δ*so*::*Hyg*^{r}; *inl*; *mat* A	NCU02794 (*so*); NCU06348 (*inl*)	Δ*so*::*Hyg*^{r}; *mat* a x *inl*; *mat* A	198	Cde
Δ*so*::*Hyg*^{r}; *pan-2*; *mat* A	NCU02794 (*so*); NCU10048 (*pan-2*)	Δ*so*::*Hyg*^{r}; *mat* a x *pan-2*; *mat* A	235	Cde
Δ*mak-1*::*Hyg*^{r}; *mat* A	NCU09842 (*mak-1*)	11320	169	Cde
Δ*ham-3*::*Hyg*^{r}; *mat* A	NCU08741 (*ham-3*)	11299	168	Cde
Δ*ham-4*::*Hyg*^{r}; *mat* A	NCU00528 (*ham-4*)	12081	179	Cde
Δ*ham-5*::*Hyg*^{r}; *mat* A	NCU01789 (*ham-5*)	15046	184	Cde
Δ*ham-6*::*Hyg*^{r}; *mat* A	NCU02767 (*ham-6*)	16993	185	Cde
Δ*ham-7*::*Hyg*^{r}; *mat* A	NCU00881 (*ham-7*)	13776	183	Cde
Δ*ham-8*::*Hyg*^{r}; *mat* A	NCU02811 (*ham-8*)	Δ*ham-8*::*Hyg*^{r}; *mat* a x mat A	224	Cde
Δ*ham-8*::*Hyg*^{r}; *mat* a	NCU02811 (*ham-8*)	17225	186	Cde

Supplementary Table 3. Primers used for amplifying Δ*so* and wild type nuclei in heterokaryons.

**Name**	**Sequence (5’->3’)**	**Purpose**
hygB_2f	CGGTTTCCACTATCGGCGAG	This amplicon is specific to the hygromycin resistance cassette in single-gene knockouts, therefore this primer pair is used to amplify only Δ*so* nuclei in the background of wild type nuclei
hygB_2r	GGGCGTATATGCTCCGCATT	
so_1f	GTACCTCCTCTTCCAGCACC	This amplicon is specific to the gene *so*, therefore this primer pair is used to amplify only wild-type nuclei in the background of Δ*so* nuclei
so_1r	CACCTTGGGGAACAGACCTT	

Supplementary References

1. Bastiaans, E., Debets, A. J. M. & Aanen, D. K. Experimental evolution reveals that high relatedness protects multicellular cooperation from cheaters. *Nat. Commun.* **7**, 11435 (2016).
