# Supplementary material for: Somatic deficiency causes reproductive parasitism in a fungus
Source: Nat Commun. 2021 Feb 4;12:783. doi: 10.1038/s41467-021-21050-5 (PMC7862218; doi:10.1038/s41467-021-21050-5)
Supplement: Supplementary file 3 — Description of Additional Supplementary Files [file 41467_2021_21050_MOESM3_ESM.pdf]

Description of Additional Supplementary Files

File Name: Supplementary Data 1
Description: The detected mutations in the evolved Neurospora crassa morphotypes relative to their respective ancestors.
