# Supplementary material for: Somatic deficiency causes reproductive parasitism in a fungus
Source: Nat Commun. 2021 Feb 4;12:783. doi: 10.1038/s41467-021-21050-5 (PMC7862218; doi:10.1038/s41467-021-21050-5)
Supplement: Supplementary file 5 — Reporting Summary [file 41467_2021_21050_MOESM5_ESM.pdf]

nature research

Corresponding author(s): Duur Aanen

Last updated by author(s): Dec 23, 2020

Reporting Summary

Nature Research wishes to improve the reproducibility of the work that we publish. This form provides structure for consistency and transparency in reporting. For further information on Nature Research policies, see our Editorial Policies and the Editorial Policy Checklist.

Statistics

For all statistical analyses, confirm that the following items are present in the figure legend, table legend, main text, or Methods section.

n/a	Confirmed	
☐	☒	The exact sample size (*n*) for each experimental group/condition, given as a discrete number and unit of measurement
☒	☐	A statement on whether measurements were taken from distinct samples or whether the same sample was measured repeatedly
☐	☒	The statistical test(s) used AND whether they are one- or two-sided<br>*Only common tests should be described solely by name; describe more complex techniques in the Methods section.*
☒	☐	A description of all covariates tested
☒	☐	A description of any assumptions or corrections, such as tests of normality and adjustment for multiple comparisons
☐	☒	A full description of the statistical parameters including central tendency (e.g. means) or other basic estimates (e.g. regression coefficient) AND variation (e.g. standard deviation) or associated estimates of uncertainty (e.g. confidence intervals)
☐	☒	For null hypothesis testing, the test statistic (e.g. *F*, *t*, *r*) with confidence intervals, effect sizes, degrees of freedom and *P* value noted<br>*Give P values as exact values whenever suitable.*
☒	☐	For Bayesian analysis, information on the choice of priors and Markov chain Monte Carlo settings
☒	☐	For hierarchical and complex designs, identification of the appropriate level for tests and full reporting of outcomes
☒	☐	Estimates of effect sizes (e.g. Cohen's *d*, Pearson's *r*), indicating how they were calculated

Our web collection on statistics for biologists contains articles on many of the points above.

Software and code

Policy information about availability of computer code

Data collection	No software was used to collect data.
Data analysis	Software used in the study was as follows: popoolation (v1.2.2), bwa-mem (v0.7.17-r1188), samtools (v1.9), VarScan (v2.4.4), vcfR (v1.8.0), IGV genome browser (v2.3.94), R (v3.6.1), ggplot2 (v3.3.0), Bio-Rad CFX Manager (v2.0), Adobe Illustrator CC (v.22.0.1).<br>Custom code to detect large indels was deposited in Github (https://github.com/ecoevomicsjoost/neurospora_pipeline).

For manuscripts utilizing custom algorithms or software that are central to the research but not yet described in published literature, software must be made available to editors and reviewers. We strongly encourage code deposition in a community repository (e.g. GitHub). See the Nature Research guidelines for submitting code & software for further information.

Data

Policy information about availability of data

All manuscripts must include a data availability statement. This statement should provide the following information, where applicable:

- Accession codes, unique identifiers, or web links for publicly available datasets
- A list of figures that have associated raw data
- A description of any restrictions on data availability

Raw sequencing data of Neurospora crassa morphotypes have been deposited in the GenBank database under BioProject accession PRJNA605104 [https://www.ncbi.nlm.nih.gov/bioproject/PRJNA605104]. Unique identifiers for the databases were obtained from Resource Identification Portal (RRID:SCR_008143 [https://scicrunch.org/resources/Any/search?q=SCR_008143&l=SCR_008143], RRID:SCR_001372 [https://scicrunch.org/resources/Any/search?q=SCR_001372&l=SCR_001372], RRID:SCR_006013, [https://scicrunch.org/resources/Any/search?q=SCR_006013&l=SCR_006013]. Fungal Genetics Stock Center database was used to search for knockout mutants for this study [http://www.fgsc.net/fgsc/search_form.php]. Gene nomenclature was followed from FungiDB [https://fungidb.org/fungidb/app/].

Field-specific reporting

Please select the one below that is the best fit for your research. If you are not sure, read the appropriate sections before making your selection.

- [x] Life sciences
- [ ] Behavioural & social sciences
- [ ] Ecological, evolutionary & environmental sciences

For a reference copy of the document with all sections, see nature.com/documents/nr-reporting-summary-flat.pdf

Life sciences study design

All studies must disclose on these points even when the disclosure is negative.

Sample size	No sample size calculation was performed. We followed the logic from our previous experiments [Bastiaans et al. Experimental evolution reveals that high relatedness protects multicellular cooperation from cheaters" Nat. Commun. 7, 11435 (2016)], where we showed that three biological replications is sufficient to detect differences in both competitiveness, spore yield, and allele frequency of various Neurospora strains. Hence, in the current study, for the competition experiments as well as for spore yield and allele frequency measurements of heterokaryons we used three biological replicates. To find the benefit in a heterokaryon (Figure 3), we increased number of replicates to ten (five colonies for each of the two marker combinations), because we expected more stochasticity of nuclei flow in each individual heterokaryon colony.
Data exclusions	No data were excluded.
Replication	We repeated the experiment where we measured competitiveness of soft mutant (Figure 1) with narrower range of cheater frequencies (results provided in the Figure 1 inserts), which yielded similar results.
Randomization	Most experiments were done in glass tubes or Petri plates, which were placed in random order in the rack (or in a plastic box in case of plates) and incubated. Individual heterokaryotic colonies were picked randomly from Petri plates.
Blinding	To determine competitiveness of Neurospora mutants at various frequencies as well as frequency of alleles in heterokaryons, we used phenotype counts done at once without knowing the group allocation, which was checked after the counts have been made. For the rest of experiments we were not blinded to group allocations due to nature of experiments.

Reporting for specific materials, systems and methods

We require information from authors about some types of materials, experimental systems and methods used in many studies. Here, indicate whether each material, system or method listed is relevant to your study. If you are not sure if a list item applies to your research, read the appropriate section before selecting a response.

Materials & experimental systems

n/a	Involved in the study	
[x]	[ ]	Antibodies
[x]	[ ]	Eukaryotic cell lines
[x]	[ ]	Palaeontology and archaeology
[x]	[ ]	Animals and other organisms
[x]	[ ]	Human research participants
[x]	[ ]	Clinical data
[x]	[ ]	Dual use research of concern

Methods

n/a	Involved in the study	
[x]	[ ]	ChIP-seq
[x]	[ ]	Flow cytometry
[x]	[ ]	MRI-based neuroimaging
